# Submillimeter alignment of more than three contiguous vertebrae in spinal SRS/SBRT with 6‐degree couch

**DOI:** 10.1002/acm2.12153

**Published:** 2017-08-08

**Authors:** Xin Wang, Zhongxiang Zhao, Dershan Luo, James N. Yang, Jinzhong Yang, Eric L. Chang, Paul D. Brown, Jing Li, Mary F. McAleer, Amol J. Ghia

**Affiliations:** ^1^ Department of Radiation Physics The University of Texas MD Anderson Cancer Center Houston TX USA; ^2^ Departments of Radiation Oncology University of Southern California Keck School of Medicine Norris Cancer Hospital Los Angeles CA USA; ^3^ Departments of Radiation Oncology Mayo Clinic Rochester MN USA; ^4^ Department of Radiation Oncology The University of Texas MD Anderson Cancer Center Houston TX USA

**Keywords:** 6D couch, alignment, multilevel target, SBRT, spinal SRS

## Abstract

The purpose of this study is to identify regions of spinal column in which more than three contiguous vertebrae can be reliably and quickly aligned within 1 mm using a 6‐degree (6D) couch and full body immobilization device. We analyzed 45 cases treated over a 3‐month period. Each case was aligned using ExacTrac x‐ray positioning system with integrated 6D couch to be within 1° and 1 mm in all six dimensions. Cone‐Beam computed tomography (CBCT) with at least 17.5 cm field of view (FOV) in the superior–inferior direction was taken immediately after ExacTrac positioning. It was used to examine the residual error of five to nine contiguous vertebrae visible in the FOV. The residual error of each vertebra was determined by expanding/contracting the vertebrae contour with a margin in millimeter integrals on the planning CT such that the new contours would enclose the corresponding vertebrae contour on CBCT. Submillimeter initial setup accuracy was consistently achieved in 98% (40/41) cases for a span of five or more vertebrae starting from T2 vertebra and extending caudally to S5. The curvature of spinal column along the cervical region and cervicothoracic junction was not easily reproducible between treatment and simulation. Fifty‐seven percent (8/14) of cases in this region had residual setup error of more than 1 mm in nearby vertebrae after alignment using 6D couch with image guidance. In conclusion, 6D couch integrated with image guidance is convenient and accurately corrects small rotational shifts. Consequently, more than three contiguous vertebrae can be aligned within 1 mm with immobilization that reliably reproduces the curvature of the thoracic and lumbar spinal column. Ability of accurate setup is becoming less a concern in limiting the use of stereotactic radiosurgery or stereotactic body radiation therapy to treat multilevel spinal target.

## 1 INTRODUCTION

Spinal stereotactic radiosurgery (SRS) or stereotactic body radiation therapy (SBRT) delivers high dose in one or a few fractions is increasingly used to maximize local tumor control and pain relief.^1^ One question in patient eligibility of spinal SRS/SBRT is how many contiguous spine vertebrae can be treated. In RTOG 0631 clinical trial, the limit was set to two. In our institution's current practice, it has been mostly limited to three or less contiguous vertebrae. Such a limitation is due to the caution and uncertainties of normal tissue tolerance in spinal SRS/SBRT and great concern on the technique ability to align long/large target accurately and reliably. One characteristic of spinal SRS/SBRT plans is the sharp drop‐off of dose between the target and spinal cord due to the close proximity of this critical structure to the target lesion. Even a small setup error of 2 mm or 2° can significantly affect both the target and spinal cord dose.^2^ The effect of small rotational error is more significant when treating large target spanning three or more vertebral bodies, since such error could cause large displacement at distance from the isocenter. Rotational error is especially challenging to correct with a conventional treatment couch.

Recent advance in 6‐degree (6D) couches has made it easier to quickly and accurately correct both rotational and translational shifts. Hypothetically, if the curvature of the vertebral column could be reproduced during treatment as the simulation, the 6D couch would be able to accurately align several contiguous vertebrae under image guidance. The purpose of this work is to assess how many contiguous vertebrae can be reliably aligned within 1 mm using full body immobilization and ExacTrac x‐ray (Brainlab) positioning system with its integrated 6D couch. In the study, we examined the largest residue error of each vertebra by replicating the process of CBCT verification of the ExacTrac alignment in the Pinnacle treatment planning system (TPS). This is different than most registration software, such as the online ExacTrac and Varian system, which only display the residual error as a 3D or 6D shift of isocenter. Our result can be easily used to estimate the dose perturbation to the target and normal tissue based on the dosimetric characteristic of spinal SRS/SBRT plan, hence the feasibility of treating a long target. We hypothesize that, with appropriate immobilization, image guidance, and the adjustments made feasible by the 6D couch, spinal SRS/SBRT may be utilized to treat to a larger population of patients with contiguous, multilevel metastatic disease to the spine. In addition to the setup uncertainties, this study also examined two real patient cases treating four and five contiguous vertebrae. The dosimetric parameters including both maximum dose to a point (Dmax) and maximum dose to a partial volume of 1, 2, and 5 cm^3^ (D1 cc, D2 cc and D5 cc) and their association with added volume are discussed.

## 2 METHODS AND MATERIALS

### 2.A Patient selection and immobilization

All spinal SRS/SBRT patient treatments from January 1 to March 31 of 2016 were analyzed in this retrospective study following institution IRB‐approved protocol. This included a total of 45 cases with each case treated with one isocenter. The vertebral targets treated at each case are detailed in Table 1 as lesion site ranging from C1 to S3. The immobilization devices for each patient were made during CT simulation process. The primary immobilization device for each patient is a full body vacuum cradle (BlueBAG, Elekta). The cradle was custom molded to the patient's body to provide reproducibility, stability, and comfort of patient positioning. In addition, a thermoplastic mask (High Precision System for Head, Neck and Shoulders, Orfit) or plastic body cover sheet (BodyFIX, Elekta) was added for precise patient positioning and immobilization, depending on spine level to be treated. The thermoplastic mask which wrapped around the shoulder and covered most of the chest area was used for all C‐spine and most of upper T‐spine (T1–T5) patients. A body cover sheet with vacuum seal was used on lower T‐, L‐, and S‐spine patients. Each patient's head rested on either a standard headrest or custom made one with a cushion (Klarity AccuCushions, Klarity Medical Products), which wraps around the posterior part of the head and neck. The details of immobilization setup of each patient are also summarized in Table 1. Following the generation of each patient's specific immobilization devices, a planning CT was acquired with 1 mm slice thickness, ranging at least 10 cm above or below the target to be treated. The resolution of X and Y directions in each slice was also 1 mm.

**Table 1:** List of cases, lesion locations, and immobilization setup. “x” marks the immobilization device was used

Case #	Lesion site	Immobilization				
		BlueBAG cradle	BodyFix cover sheet	Orfit mask	Standard headrest	Klarity headrest
1	C1	X		X		X
2	C1	X		X		X
3	C4	X		X	X	
4	C5	X		X	X	
5	C5‐7	X		X		X
6	C7	X		X		X
7	C7	X		X	X	
8	T1	X		X		X
9	T1‐3	X		X		X
10	T2‐3	X		X	X	
11	T3‐4	X		X		X
12	T3‐4	X		X	X	
13	T4	X		X		X
14	T4	X		X		X
15	T5	X		X	X	
16	T5‐8	X	X		X	
17	T6	X	X		X	
18	T6	X	X		X	
19	T6‐7	X	X		X	
20	T6‐7	X	X		X	
21	T8	X	X		X	
22	T8‐10	X	X		X	
23	T9	X	X		X	
24	T9‐10	X	X		X	
25	T10	X	X		X	
26	T10‐11	X	X		X	
27	T11	X	X		X	
28	T12	X	X		X	
29	T12	X	X		X	
30	T12	X	X		X	
31	T12‐L2	X	X		X	
32	L1	X	X		X	
33	L1	X	X		X	
34	L1‐2	X	X		X	
35	L2	X	X		X	
36	L2‐3	X	X		X	
37	L2‐4	X	X		X	
38	L4	X	X		X	
39	L4	X	X		X	
40	L2‐S1	X	X		X	
41	L5	X	X		X	
42	L5	X	X		X	
43	L5‐S1	X	X		X	
44	S1	X	X		X	
45	S2‐S3	X	X		X	

### 2.B Multimodality imaged‐guided alignment in patient treatment setup

Each case was treated in a suite equipped with a Varian TrueBeam Stx linear accelerator (LINAC) and ExacTrac x‐ray patient positioning system with an integrated 6D couch. The TrueBeam on board image (OBI) system has the ability to perform kV, MV, and CBCT. ExacTrac uses high‐resolution stereoscopic x‐ray images to detect patient position and an infrared (IR) optical system with 6D couch to apply shifts to align patient position in six dimensions. The image isocenters (kV, CBCT, and ExacTrac) and radiation isocenter congruence were tested bi‐weekly following a quality assurance procedure, which first aligned a tungsten ball (6.5 mm in diameter) to the kV (CBCT) isocenter. The ExacTrac isocenter relative to the ball was acquired through the Winston‐Lutz test in the ExacTrac software. The radiation isocenter relative to the ball was measured using MV portal images (gantry at 0, 90, 180, and 270) of a 5 × 5 cm MLC field and an algorithm described in the literature.^3^ The discrepancies between isocenters were calculated from their positions relative to the ball. They were reliably within 0.5 mm, with no need of recalibration of image isocenters over a 3‐year period. The only time an image isocenter calibration maybe required was when the x‐ray source was serviced. Daily ExacTrac test was also performed with manufacture provided calibration/daily phantom to verify the IR optical system with 6D couch had submillimeter accuracy.

**Figure 1:**
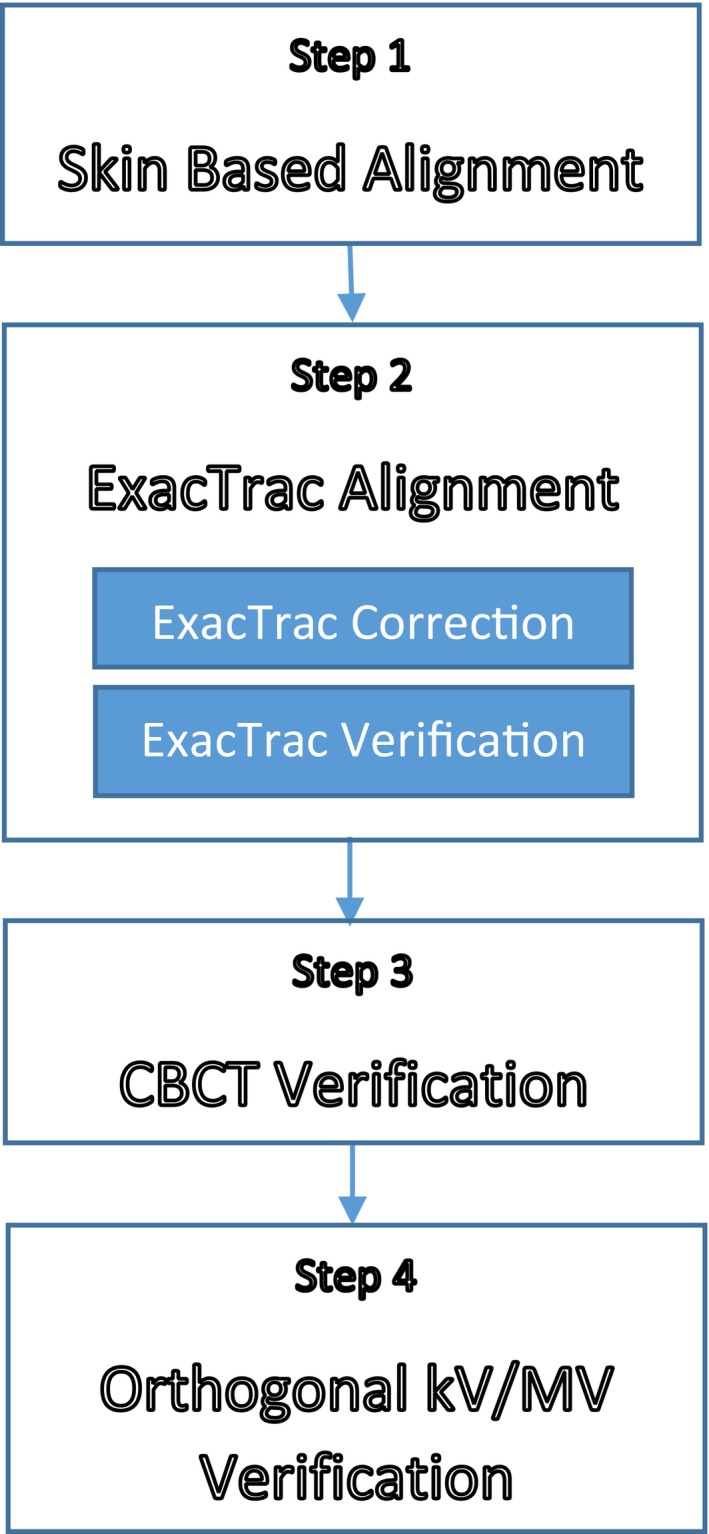
Major steps and image modalities in the work flow of our initial setup.

The goal of the initial setup accuracy in our spinal SRS/SBRT treatment is to achieve accuracy of <1 mm and <1° in all six dimensions. Figure 1 shows the diagram of major steps and image modalities used in the initial setup process to achieve this goal. This process is summarized here: (a) The patient was first settled into the immobilization apparatus and aligned using skin marks (such as leveling/rotation marks, marked isocenter, or ExacTrac infrared spheres). This step aimed to reproduce the body position as close as the simulation and eliminate large rotational and translational error. (b) Following skin‐based setup, ExacTrac positioning system was used as the primary tool to align target through couch shifting (referred as “ExacTrac Correction” in the diagram). In the “ExacTrac Correction” process, the x‐ray system and its image registration software were used to detect shifts in 6D freedom, and shifts were applied automatically using 6D couch under the guidance of its optical system. After correction, the position was verified with ExacTrac x‐ray system and its image registration software. The verification was considered a success if the residual error displayed in ExacTrac software was within 1 mm and 1° in all six dimensions. (c) A CBCT was performed immediately after ExacTrac verification passed initial setup criteria. Its resolution matched the planning CT in superior‐anterior direction and was slightly better in two other directions. The CBCT was used as the gold standard in the multimodality image guidance process to verify the accuracy of alignment by ExacTrac. Clinicians used the online software at the LINAC image console to verify each slice over the region of interest has residual error within 1 mm between CBCT and planning CT. (d) Once the CBCT was deemed to be in agreement with ExacTrac, kV/MV orthogonal images and another ExacTrac image verification were taken to verify all imaging modalities were in agreement the setup accuracy meeting the predetermined criteria, and intrafraction motion was not an issue through the lengthy initial setup process.

### 2.C Vertebrae alignment accuracy analysis

ExacTrac alignment and CBCT verification as prescribed in the previous section are the two steps relevant to this study. During treatment, only the alignments of vertebrae with disease were examined. This limited the number of contiguous vertebrae to three or less for the majority of cases as detailed in Table 1. In this study, we assessed all vertebrae visible on CBCT by replicating the process of CBCT verification of the ExacTrac alignment in the Pinnacle treatment planning system (TPS). This was performed in TPS by aligning the image center of the CBCT to the isocenter on planning CT. This approach, which did not involve any rotational and translational correction through image registration, preserved the residual error after patient alignment with ExacTrac. It was a true replication of display CBCT relative to the planning CT as in step 3 of the initial setup process.

**Figure 2:**
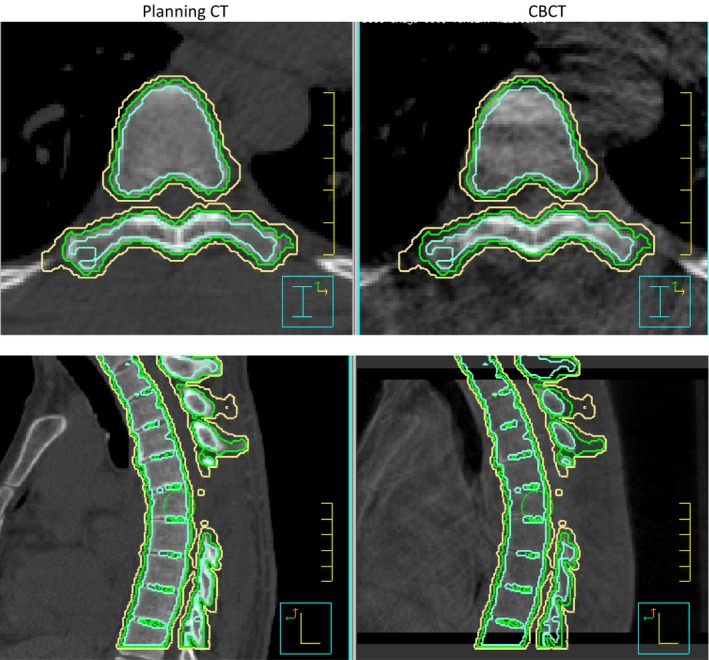
Residual error analysis for spinal column (T3‐9) of case # 16. Planning CT is on the left side and CBCT is on the right side. Green is the auto‐contour of vertebrae. Cyan and yellow are 1 mm contraction and expansion of vertebrae in planning CT. The axial and sagittal slice demonstrates the residual error is less than 1 mm for vertebrae T3‐9.

Most registration software, such as the online ExacTrac and Varian system and Pinnacle TPS, only display the residual error as a 6D shift of isocenter after performing rigid registration. Such information may be used to calculate the residual error of a point at a particular location. But it can be troublesome for long target involving multiple vertebrae, which could have curvature change. In this study, we analyzed the residual setup error of each individual vertebra (VB). The analysis was performed in TPS by expanding and contracting the VB contour with a margin in integral millimeter values on the planning CT such that the new contour would be large enough to enclose the corresponding vertebra contour on CBCT. The margin of the expansion was considered the setup error of the VB. This setup error reflected the largest residual error of each individual VB. Figure 2 demonstrates the setup error analysis for case # 16, with green, yellow, and cyan being the contours of VB, VB+1 mm, and VB‐1 mm, respectively. This figure shows 7 VB on CBCT are enclosed in VB+1 mm and VB‐1 mm contours. Therefore, the setup error for seven vertebrae in this case is within 1 mm. Similar analyses were performed on the CBCT data from the 1st fraction treatment of all 45 cases in the study.

### 2.D Treatment planning and dosimetric analysis of long target patient cases

**Figure 3:**
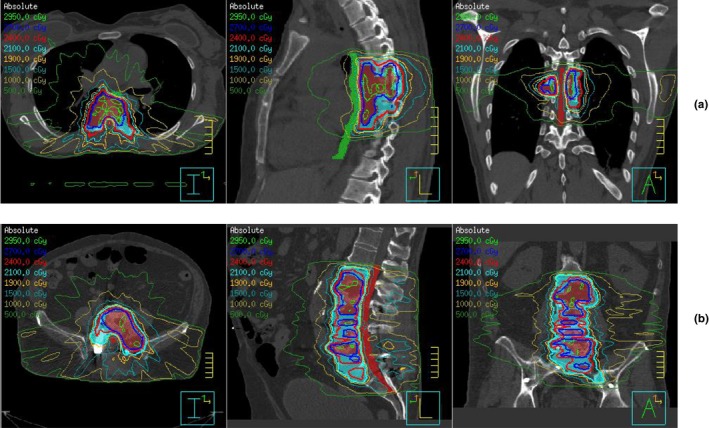
Treatment plans for case # 16 (a) and # 40 (b). The graphs display both structures and isodose lines of the clinical plans. The structures include both targets (GTV in maroon, CTV in sky blue) and critical structures (spinal cord/cauda equina in red, esophagus is green) in close proximity.

In this study, two cases were treated with more than three contiguous vertebrae. Case # 16 was a melanoma patient with disease from T5 to T8 level. Figure 3(a) shows the location of gross tumor volume (GTV) and clinical target volume (CTV) from T5 to T8 level. The challenge for this case included spinal cord at T6 level which was right next to the GTV, and more significantly, esophagus which was just anterior of GTV over all four levels from T5 to T8. Case # 40 is a patient with metastatic renal cell carcinoma. He had a resection at L5, but progressed with new disease from L2 to S1, as shown in Fig. 3(b). The major challenge for this case was the cauda equina which was surrounded by the target over five contiguous vertebrae. As part of our initial experience, both treatments were planned for 27 Gy in three fractions, instead of single fraction treatment. The planning followed our standard practice of spinal SRS/SBRT planning,^4^ which used nine to 11 posterior beams to generate step‐and‐shoot IMRT plan. Critical normal tissues were contoured to 10 mm above and below CTV levels. The constraints of nearby critical normal tissues specified by the physicians are listed in Table 2. The dosimetric value achieved by the clinical plan and the perturbations to them due to potential small alignment error were calculated with a 1 and 2 mm vertical isocenter shifts, which was in a sharp dose drop‐off direction. To investigate the dosimetric impact of long target to the critical structures, we divided the critical structure volumes into sub volumes with each sub volume corresponding to a vertebra level, and examined the effects of extra volumes on point dose Dmax (maximum dose to 0.01 cm^3^) and partial volume dose of D1 cc, D2 cc, and D5 cc.

**Table 2:** Dose constraints of critical structures near two long target cases. It includes both the dosimetric values specified by the physician and achieved by the clinical plan assuming 0, 1, and 2 mm misalignment

Case #	Normal tissue	Constraints from physician	Clinical plan
16	Spinal cord	Dmax ≤18 Gy D1 cc ≤14 Gy	Dmax = 16.7 Gy D1 cc = 13.8 Gy
	Esophagus	Dmax ≤24 Gy D1 cc ≤18 Gy	Dmax = 22.5 Gy D1 cc = 17.5 Gy
40	Cauda equina	Dmax ≤21 Gy D2 cc ≤18 Gy	Dmax = 20.9 Gy D2 cc = 17.8 Gy

## 3 RESULTS

### 3.A Vertebrae alignment accuracy analysis

**Figure 4:**
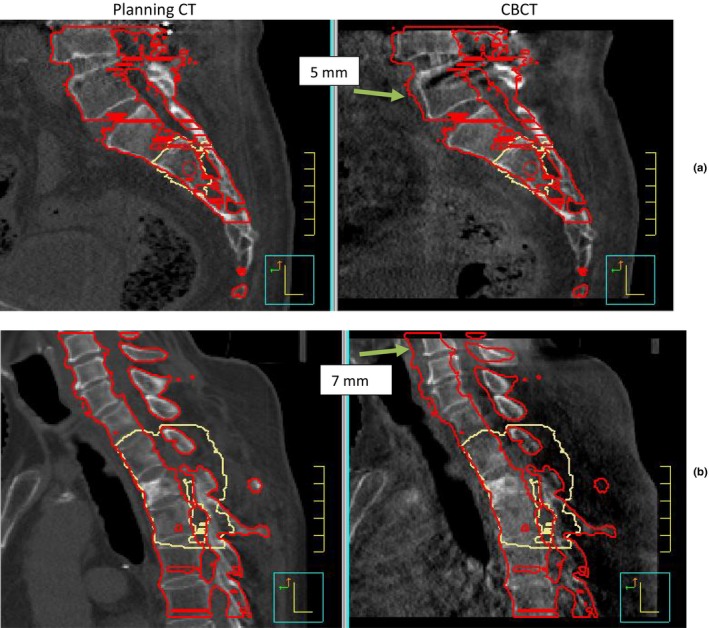
Sagittal view of case # 45 (a) and # 10 (b). Red and yellow are contours of vertebrae and targets in planning CT. Large residual errors due to curvature change at L to S transition (a) and C to T transition (b) are marked with arrows.

The CBCT had a range of at least 17.5 cm in the superior–inferior direction in the study. This scan covered five to nine contiguous vertebrae depending on the patient size, disease location, and spinal column curvature. For each case treated, the residual error of each individual vertebra visible is marked by an integer in Table 3. A number “1” means the corresponding vertebra was aligned accurately within 1 mm, “2” was aligned within 2 mm, and so on for other numbers. Cases in Table 3 with residual setup error of more than 1 mm are marked in red to identify regions of the spinal column with difficulties meeting the submillimeter accuracy. Target areas were marked as yellow cells in Table 3. Starting with case # 5, there were 41 cases, in which the CBCT displayed a vertebra between T2 and S5. Among them, four cases (case # 5 to 8) showed initial setup accuracy can be achieved within 1 mm for a span of five or more vertebrae centered on T2 vertebra. Another 36 cases (case # 9 to 44) showed initial setup accuracy can be achieved within 1 mm for a span of five or more vertebrae starting from T2 vertebra extending to more caudal vertebra levels. Case # 45 was the only exception. Figure 4(a) shows that curvature at L‐ to S‐spine transition changed during treatment relative to simulation for this case, which caused the L5 to be off by 5 mm while targets (S2–S3) were aligned within 1 mm. Lower C‐spine and the transition to T‐spine was identified as being a difficult region for submillimeter setup accuracy, as all the red cells except case # 45 were located in this region. Of 14 cases that involved part of C1 to T1 region (case # 1 to 14), eight cases had vertebrae with residual setup error of more than 1 mm. This error can be as high as 7 mm at the distal end from the isocenter as demonstrated in Fig. 4b.

**Table 3:** Residual error in millimeter integrals of vertebrae visible in the CBCT of each case

#	C1	C2	C3	C4	C5	C6	C7	T1	T2	T3	T4	T5	T6	T7	T8	T9	T10	T11	T12	L1	L2	L3	L4	L5	S1	S2	S3	S4	S5
1	1	1	1	2	3																								
2	1	1	1	1	3																								
3	1	1	1	1	1	1	1	1																					
4		1	1	1	1	1	2	2																					
5		2	1	1	1	1	1	1	1	1	1																		
6			4	3	2	1	1	1	1	1																			
7			5	5	3	1	1	1	1	1	1																		
8			1	1	1	1	1	1	1	1	1	1																	
9					1	1	1	1	1	1	1	1	1																
10						7	5	2	1	1	1	1	1																
11							1	1	1	1	1	1	1																
12							1	1	1	1	1	1	1																
13						4	3	1	1	1	1	1	1	1															
14					1	1	1	1	1	1	1	1	1	1															
15									1	1	1	1	1	1	1														
16										1	1	1	1	1	1	1													
17										1	1	1	1	1	1	1													
18										1	1	1	1	1	1	1													
19										1	1	1	1	1	1	1													
20											1	1	1	1	1	1													
21												1	1	1	1	1	1												
22													1	1	1	1	1	1	1										
23													1	1	1	1	1	1	1										
24														1	1	1	1	1	1										
25														1	1	1	1	1	1										
26															1	1	1	1	1	1									
27																1	1	1	1	1	1								
28																	1	1	1	1	1								
29																	1	1	1	1	1								
30																	1	1	1	1	1								
31																		1	1	1	1	1							
32																		1	1	1	1	1							
33																	1	1	1	1	1	1	1						
34																		1	1	1	1	1	1						
35																			1	1	1	1	1						
36																			1	1	1	1	1						
37																				1	1	1	1	1					
38																					1	1	1	1	1				
39																					1	1	1	1	1	1			
40																					1	1	1	1	1				
41																						1	1	1	1	1	1		
42																						1	1	1	1	1	1	1	
43																						1	1	1	1	1	1		
44																							1	1	1	1	1	1	
45																								5	1	1	1	1	1

Using the data in Table 3, statistical analysis was performed to evaluate the possibility of submillimeter alignment of three to five contiguous vertebrae in various region of spinal column. When the location of a single target vertebra (STV) is categorized into two groups, C (C‐spine) and TLS (T‐, L‐, and S‐ spine), the statistical analysis shows a clear separation in the possibility of submillimeter alignment of five (STV + 2 above and 2 below) contiguous vertebrae, 42.9% for the C region and 97.4% for the TLS region. This difference is significant with *P* < 0.001 in chi‐square test. For three contiguous vertebrae (STV + 1 above and 1 below), submillimeter alignments are achievable in all regions.

### 3.B Treatment planning and dosimetric analysis of long target patient cases

Following our standard practice of spinal SRS/SBRT planning, step‐and‐shoot IMRT plans were successfully optimized to meet all dose constraints requested by the physician for two long target cases (Table 2). The isodose lines of the clinical plans for case # 16 and # 40 are shown in Figs. 3(a) and 3(b), respectively. They illustrated the sharpest dose drop‐off around the nerve structure (spinal cord/cauda equina) and esophagus. Tables 3, 4, 5 lists Dmax, D1 cc, D2 cc, and D5 cc to both the whole and sub volumes of critical structures. In these tables, values for misalignment of 0 mm are from the clinical plan, which was likely achieved with <1 mm setup error during delivery. Values of 1 and 2 mm misalignments were assumed and calculated with a 1 and 2 mm isocenter vertical shift. They were used to estimate the dose perturbation which was calculated as the average value changes from 0 to 1 and 1 to 2 mm misalignments.

**Table 4:** Dosimetric values to the spinal cord of case # 16. The spinal cord volumes include the total volume (T5 to T8) over the target area and sub volume in each individual vertebra level. Misalignment is a vertical shift in isocenter of clinical plan. Dose pertubation is the average of dose value changes from 0 to 1 and 1 to 2 mm misalignment

Vertebrae level	Spinal cord volume (cm^3^)	Misalignment (mm)	D_max_ (Gy)	D_1 cc_ (Gy)	D_2 cc_ (Gy)
T5	0.90	0	16.3		
		1	16.9		
		2	18.4		
		Dose perturbation (Gy/mm)	0.6		
T6	0.99	0	16.7		
		1	18.8		
		2	21.2		
		Dose perturbation (Gy/mm)	2.2		
T7	0.98	0	15.0		
		1	15.9		
		2	17.8		
		Dose perturbation (Gy/mm)	0.9		
T8	0.98	0	14.9		
		1	14.8		
		2	14.7		
		Dose perturbation (Gy/mm)	−0.1		
T5 to T8	3.85	0	16.7	13.8	12.6
		1	18.8	14.0	13.0
		2	21.2	14.3	13.4
		Dose perturbation (Gy/mm)	2.2	0.3	0.4

**Table 5:** Dosimetric values to the esophagus of case # 16. The esophagus volumes include the total volume (T5 to T8) over the target area and sub volume in each individual vertebra level. Misalignment is a vertical shift in isocenter of clinical plan. Dose pertubation is the average of dose value changes from 0 to 1 and 1 to 2 mm misalignment

Vertebrae level	Esophagus volume (cm^3^)	Misalignment (mm)	D_max_ (Gy)	D_1 cc_ (Gy)	D_2 cc_ (Gy)	D_5 cc_ (Gy)
T5	1.07	0	22.2	12.3		
		1	23.7	12.8		
		2	25.0	13.3		
		Dose perturbation (Gy/mm)	1.4	0.5		
T6	1.81	0	21.3	14.3		
		1	23.1	15.2		
		2	25.0	16.2		
		Dose perturbation (Gy/mm)	1.9	1.0		
T7	2.12	0	21.1	14.9	11.1	
		1	22.7	16.1	11.9	
		2	24.4	17.6	12.8	
		Dose perturbation (Gy/mm)	1.7	1.4	0.9	
T8	2.37	0	21.2	13.6	10.5	
		1	22.7	14.3	11.1	
		2	24.5	15.3	11.7	
		Dose perturbation (Gy/mm)	1.7	0.9	0.6	
T5 to T8	7.37	0	22.5	17.5	16.1	13.2
		1	24.0	19.0	17.3	14.0
		2	25.7	20.6	18.8	14.9
		Dose perturbation (Gy/mm)	1.6	1.6	1.4	0.9

**Table 6:** Dosimetric values to cauda equina of case # 40. The cauda equina volumes include the total volume (L2 to S1) over the target area and sub volume in each individual vertebra level. Misalignment is a vertical shift in isocenter of clinical plan. Dose pertubation is the average of dose value changes from 0 to 1 and 1 to 2 mm misalignment

Vertebrae level	Cauda equina volume (cm^3^)	Misalignment (mm)	D_max_ (Gy)	D_1 cc_ (Gy)	D_2 cc_ (Gy)	D_5 cc_ (Gy)
L2	6.33	0	20.6	17.4	16.5	13.6
		1	21.2	18.0	17.0	14.1
		2	22.0	18.7	17.5	14.6
		Dose perturbation (Gy/mm)	0.7	0.7	0.5	0.5
L3	5.77	0	20.2	17.0	15.8	12.4
		1	21.2	17.6	16.5	12.8
		2	22.3	18.5	17.3	13.3
		Dose perturbation (Gy/mm)	1.1	0.8	0.8	0.5
L4	5.43	0	19.4	16.4	15.5	11.7
		1	20.5	17.1	16.0	12.1
		2	21.6	18.0	16.5	12.6
		Dose perturbation (Gy/mm)	1.1	0.9	0.5	0.5
L5	7.70	0	20.4	16.8	15.7	13.2
		1	21.3	17.5	16.3	13.5
		2	22.4	18.4	17.0	13.9
		Dose perturbation (Gy/mm)	1.0	0.8	0.7	0.4
S1	10.64	0	20.6	16.3	15.4	13.3
		1	21.2	16.6	15.7	13.6
		2	22.6	17.1	16.0	14.0
		Dose perturbation (Gy/mm)	1.0	0.4	0.3	0.4
L2 to S1	35.87	0	20.9	18.5	17.8	16.9
		1	21.7	19.1	18.5	17.4
		2	22.7	20.0	19.4	18.2
		Dose perturbation (Gy/mm)	1.0	0.8	0.8	0.7

Table 3 includes the data for spinal cord of case # 16. It shows the spinal cord volume at T6 level is the one responsible for the Dmax to the whole spinal cord volume. Spinal cord volume at this level has the highest Dmax (16.7 Gy from clinical plan) and the sharpest dose drop, in which a 1 mm vertical misalignment could cause ~2.2 Gy dose perturbation to the Dmax received the spinal cord. In other three levels, Dmax to spinal cord volume is lower and dose perturbation is <1 Gy/mm, since spinal cord is further away from GTV at these levels than T6 level. D1 cc and D2 cc are not an issue at each individual level, since the spinal cord volume at each level is less than 1 cc. As more spinal cord is receiving dose with longer target, these values increase. Considering all four vertebrae levels, the D1 cc and D2 cc to the spinal cord volume are 13.8 and 12.6 Gy from the clinical plan. Dose perturbations due to misalignment to these values are small (0.3 to 0.4 Gy/mm).

Table 4 shows the data for esophagus of case # 16. Unlike the spinal cord, the dose distribution around esophagus at different vertebral levels is similar in this case, since the esophagus has similar distance to GTV in all four vertebrae levels. This is reflected on the Dmax of clinical plan in Table 4 which has values of 21.1 to 21.3 Gy from T6 to T8 and a slightly higher value of 22.2 Gy at T5. The dose perturbation to Dmax is also similar (1.4 to 1.8 Gy/mm) though all four levels. D1 cc, D2 cc, and D5 cc increase with more esophagus volume receiving dose, and they are at 17.5, 16.1 and 13.2 Gy respectively for the clinical plan to the whole esophagus volume. The dose perturbations to them are 1.6, 1.4, and 0.9 Gy/mm, respectively.

Table 5 shows the data for cauda equina of case # 40. In this case, dose distribution around cauda equina is consistent at all five levels with Dmax within a range of 20.2 to 20.6 Gy at each individual level. Dmax is slightly higher at 20.9 Gy for the whole cauda equina. The dose drop‐off is also similar at each individual level at ~1 Gy/mm. D1 cc, D2 cc, and D5 cc are available at each individual level in this case. At each individual level, they average 16.8, 15.8, and 12.8 Gy and increase to 19.1, 18.5, and 17.4 Gy, respectively for the whole volume. The dose perturbation to them is 0.7 to 0.8 Gy/mm for the whole volume.

## 4 DISCUSSION

During spinal SRS/SBRT treatment, it is common to encounter small rotational errors close to 2° in any of the three directions (pitch, roll, and yaw) after skin‐based setup. A 6D couch allows for convenient correction of rotational shifts by moving the couch in a controlled way. In contrast, it is very difficult to address rotational setup errors with a conventional couch, which requires patient position to be adjusted in a “trial‐and‐error” fashion. To utilize 6D couch for accurate multiple‐level vertebrae alignment, the curvature of the spinal column has to be reproducible, since 6D couch corrects the rotational error through rigid couch movement. Our results demonstrated the ability of ExacTrac with 6D couch adjustment to correct such rotational error accurately, and the excellence of full body cradle to reproduce the patient position and spinal curvature from T2 to S5. The patients treated to targets involving the C‐spine to upper T‐spine were mainly supported by a head rest with air filling the gap between the headrest and cradle, which, in part, made it difficult to reproduce the curvature in this region. This setup variability may not be clinically significant when aligning a small target spanning one or two contiguous vertebrae, but can be a challenge in safely and effectively treating long targets covering three or more continuous vertebrae. A custom made headrest that fills the empty air space to provide continuous support of head and neck (HN) region to the underlying cradle is believed to help reproducing the curvature in the C‐ and upper T‐spine region. Wang et al. reported a customized head and shoulder with Klarity AccuCushion helped reproducing the positioning of HN region.^5^ However, our data showed cases with setup issues in this region are about equal between standard and customized headrests. The use of customized headrest is still a learning process for us and worth a future study.

Our study focused on the initial geometric alignment of each vertebra over a long column. More importantly, the knowledge gained here enabled us to evaluate the likely dose perturbation to the nearby normal tissues during delivery.

Spinal cord is of greatest concern due to its close proximity to the target and high consequence of injury to this structure. The risks of radiation myelopathy in spinal SRS/SBRT were very well studied^6^, ^7^, ^8^, ^9^, ^10^, ^11^, ^12^, ^13^, ^14^, ^15^, ^16^, ^17^ with Dmax been generally accepted as the parameter of dosimetric constraint. Kirkpatrick et al. summarized nine published reports of spinal cord dose and myelopathy in 1,400 patients who received spinal SRS/SBRT.^16^ They recommended a maximum cord dose of 13 Gy in a single‐fraction or 20 Gy in three‐fractions to limit the risk of spinal cord injury to <1%. One of the significant challenge in spinal treatment planning is maximizing the dose and coverage to the target while maintain Dmax to the spinal cord under a safe value. In IMRT treatment planning, this is dictated by the location of target relative to the spinal cord, not the volume associated with extra vertebrae. Case # 16 demonstrated a single vertebra region was responsible for the Dmax, while spinal cord in this region is much closer to the GTV than other regions. Multiple vertebrae regions could contribute to Dmax, as in the case # 40. However, if the extra vertebrae can be quickly set up as acutely as one or two vertebrae during treatment, spinal cord with longer target is as safe short target. In our practice, 2 mm is used as a safety margin to evaluate potential dose perturbation to the Dmax of spinal cord. Same margin can be used for long target in the non C‐spine region, as result in this study shows T, L, and S spine has reliable submillimeter setup accuracy for multi (>3) vertebrae target.

Esophagus is another organ in close proximity to the targets involving the lower C‐ and T‐spine. Comparing to spinal cord, the dose constraints to the esophagus in spinal SRS/SBRT are less understood. Even though the dose constraint standard has yet been established for esophagus, partial volume constraints, such as dose to a small volume of 1 to 5 cc (D1 cc to D5 cc), and Dmax have been suggested in previous reports and used in clinical trials.^18^, ^19^, ^20^ Unlike a point dose, such as Dmax to the cord, which is unlikely to be effected by the extra vertebrae, a partial volume parameter, like D1 cc, generally increases with move critical structure volume receiving dose. This was demonstrated by the esophagus and cauda equina dose in the study. This study also showed the dose perturbation to 7.37 cc of esophagus due to misalignment is ~1.6 G/mm for D1 cc and decrease to 0.9 Gy/mm for D5 cc. These numbers could be significantly different depending on the volume and the dose drop‐off demands around the critical structure. As a result, more attention is needed when considering partial volume constraints in planning for long target.

There are other concerns in treating long target, particularly, the accuracy of target and critical structure contours. MRI imaging is used in spinal SRS/SBRT to accurately delineate gross disease and the spinal cord/cauda equina. These contours are transferred onto the planning CT through image registration for treatment planning. The accuracy of these contours can be compromised by the registration process when the patient has significant difference in body posture between CT simulation and MRI imaging. In addition, the location of spinal cord and the shape of soft tissue involvement of gross disease could also be effected by the posture.^5^ Optimizing MRI imaging by scanning the patient in the custom made spinal immobilization device which reproduces the spinal column curvature as in CT could be valuable in treating multilevel targets.

## 5 CONCLUSION

Full body vacuum cradle custom molded to the patient body is effective in reproducing and maintaining patient position and spinal curvature from T‐ to S‐spine. Long thoracic and lumbar spinal targets that span more than three contiguous vertebrae in this region can be consistently aligned within 1 mm with the use of 6D couch and image guidance. With current technology, setup accuracy is not the limiting factor in treating select long targets using spinal SRS/SBRT.

## CONFLICT OF INTEREST

The authors declare no conflict of interest.
